# Low level of extra-pair paternity between nearest neighbors results from female preference for high-quality males in the yellow-rumped flycatcher (*Ficedula zanthopygia*)

**DOI:** 10.1371/journal.pone.0172713

**Published:** 2017-03-03

**Authors:** Mingju E, Ye Gong, Jiangping Yu, Siyu Zhang, Qianxi Fan, Yunlei Jiang, Haitao Wang

**Affiliations:** 1 Jilin Provincial Engineering Laboratory of Avian Ecology and Conservation Genetics, School of Life Sciences, Northeast Normal University, Changchun, China; 2 Jilin Key Laboratory of Animal Resource Conservation and Utilization, Northeast Normal University, Changchun, China; 3 Key Laboratory of Vegetation Ecology, Ministry of Education, Institute of Grassland Science, Northeast Normal University, Changchun, China; 4 Animal Scientific and Technological Institute, Agricultural University of Jilin, Changchun, China; University of Missouri Columbia, UNITED STATES

## Abstract

Extra-pair copulation is considered to be a means by which females can modify their initial mate choice, and females might obtain indirect benefits to offspring fitness by engaging in this behavior. Here, we examined the patterns of extra-pair paternity and female preferences in the yellow-rumped flycatcher *(Ficedula zanthopygia)*. We found that female yellow-rumped flycatchers are more likely to choose larger and relatively highly heterozygous males than their social mates as extra-pair mates, that the genetic similarity of pairs that produced mixed-paternity offspring did not differ from the similarity of pairs producing only within-pair offspring, and that extra-pair offspring were more heterozygous than their half-siblings. These findings support the good genes hypothesis but do not exclude the compatibility hypothesis. Most female yellow-rumped flycatchers attained extra-pair paternity with distant males rather than their nearest accessible neighboring males, and no differences in genetic and phenotypic characteristics were detected between cuckolded males and their nearest neighbors. There was no evidence that extra-pair mating by female flycatchers reduced inbreeding. Moreover, breeding density, breeding synchrony and their interaction did not affect the occurrence of extra-pair paternity in this species. Our results suggest that the variation in extra-pair paternity distribution between nearest neighbors in some passerine species might result from female preference for highly heterozygous males.

## Introduction

Genetic studies have revealed that approximately 86% of surveyed monogamous passerine species pursue extra-pair copulations as part of a mixed mating strategy [1,2]. Extra-pair copulation is considered to be a means by which females can modify their initial mate choice because they are often constrained in their choice of social mates [3–5]. Although the advantage of extra-pair copulation for females is debated, one potential benefit that females may obtain from extra-pair copulation is an indirect genetic benefit to offspring fitness, either by inheriting good genes or by gaining compatible genes from a high-quality mate [6–9]. A variety of socially monogamous passerine females actively seek extra-pair copulations [1,10–13], and there is extensive evidence that they do so for genetic benefits [14–16]. Extra-pair copulation is valuable for males that obtain extra-pair paternity because they increase their reproductive success by siring offspring outside of their pair bonds without having to provide parental care to the extra-pair young [17], but it is disastrous for males that lose paternity [18].

Males have developed pre- and post-copulatory strategies to decrease the risk of paternity loss in their own nests, such as mate guarding, frequent within-pair copulation, and direct physical punishment of the female [19–22]. The effectiveness of male paternity assurance strategies, however, can be affected by either individual female and male qualities, e.g., a male’s ability to guard and a female’s ability to evade her mate [1,23–25], or by the male’s costs of paternity assurance [26–28] and female experience [29–31]. Additionally, the effectiveness of male paternity assurance strategies can be precluded by ecological or social factors, such as intense nest site competition [32–34]. Therefore, observed patterns of extra-pair paternity in socially monogamous species may primarily reflect a balance between the costs of mates’ extra-pair behavior and female genetic benefits to offspring fitness [13,29,35].

For territorial passerines, neighboring males are potential conspecific rivals to the territory owner for resources and mates [25,36,37]. They might pose various levels of threat to the territory owners because as they are likely to show differences in competitive ability and attractiveness to females [38]. The most frequently observed extra-pair paternity distribution pattern in passerines is that neighboring males are most likely to be extra-pair sires [25,39–42]. This behavior was interpreted to result from the easy and rapid access to the mates of the neighbors living close by, especially in very sedentary species [43–45]. Although in some species, extra-pair paternity primarily occurs between nearest neighbors [46–48], in other species extra-pair paternity is seldom associated with the nearest neighbor [27,49,50]. At the extreme, the nearest neighbors are never extra-pair sires in mountain bluebirds (*Sialia currucoides*) [51]. Explanations proposed are that factors, such as local breeding density, the time window of accessibility to fertile females, the locations of resources, heterogeneity of the landscape, and/or species-specific differences in how far individuals of either sex will travel for extra-pair copulations, might have caused this pattern of extra-pair paternity [27,50,52].

Several studies have shown that females breeding in their natal neighborhood are more likely to be surrounded by close male relatives, and the females would traverse greater distances to obtain extra-pair copulations [53,54]. Females that are mated to high-quality social partners will likely need to travel farther away to find even better extra-pair males [31], and males surrounded by neighbors of lower quality can increase their opportunities to obtain extra-pair copulations [34]. Studies on extra-pair copulation have provided a great deal of evidence for female preference for high-quality males [55]. Thus, to better understand the variation in extra-pair paternity distribution between immediate neighbors in different passerine species, it is necessary to explore the relative quality of immediate neighbors and female preferences.

The yellow-rumped flycatcher *(Ficedula zanthopygia)* is a migratory species [56,57] that exhibits solitary nesting, biparental care, and widespread extra-pair paternity [58]. To examine whether females benefit indirectly from extra-pair mating behaviour in this species, we compared the genetic and phenotypic characteristics of extra-pair and cuckolded males as well as the genetic similarity between parental dyads producing extra-pair young and only within-pair young and the heterozygosity between extra-pair and within-pair offspring. To examine whether the quality of the nearest neighboring male affects female extra-pair mate choice, we compared the genetic diversity and phenotypic characteristics of cuckolded males to their nearest neighboring males and the genetic similarity among focal females to their partners (social or extra-pair). In addition, we examined the effects of breeding density and synchrony on the occurrence of extra-pair copulations. We predicted that if female yellow-rumped flycatchers try to gain indirect benefits from extra-pair copulations, they will prefer extra-pair mates that retain a higher genetic quality or compatibility than their within-pair sires; and if the quality of the nearest neighboring males fulfill their preferences, they would most likely be chosen as extra-pair fathers.

## Methods

### Ethical note

The experiments comply with the current laws of China, where they were performed. Fieldwork was carried out with permissions from the Zuojia Nature Reserve and the Forestry Bureau of Jilin Province of China (approval number: [2006]178). Experimental procedures (i.e. bird capturing, banding and blood sampling) were approved by the National Animal Research Authority in Northeast Normal University, China (approval number: NENU–20080416). All protocols followed the Guidelines for the Use of Wild Birds in Research [59].

### Study site

The study was conducted during breeding seasons from 2011 to 2013 at Zuojia Nature Reserve (126°1′-127°2′N, 44°6′-45°5′E) in Jilin Province, northeastern China. Our study plot contained approximately 450 nest-boxes per year. Nest-boxes were installed on various species of trees at 3.0–4.0 m above the ground. The distances between the nearest nest-boxes were 30–50 m. The nests were visited at 1–2 day intervals to monitor the settling order between the nearest neighbors and to determine egg-laying dates and hatching dates. We considered a box to be occupied based on the continuous presence of nest materials. Female yellow-rumped flycatchers usually lay one egg per day (clutch sizes ranged from 5–8 eggs) per season, and they usually complete nest building within 3–4 days and start to lay eggs. The geographical position of each nest was recorded using a GPS (N400PLUS, BHCnav, Beijing, China), and these data were used to calculate the linear distances between all breeding pairs in the study plot.

To reduce the likelihood of nest abandonment as a result of early interference, adults were captured during the nestling period, 6–7 days after hatching. Provisioning adults were captured at nests with nest-box traps (a small transparent plastic sheet on an internal wall to cover the nest-box entrance) or mist nets. All adults and nestlings were outfitted with aluminum bands for individual recognition. A small blood sample (approximately 20 μl) was taken from both adult and nestling individuals by puncturing the brachial vein within 3 min of capture. Blood samples were stored in absolute ethanol in the field, then stored at -20°C until the DNA was extracted. Parent birds were measured with a caliper to the nearest 0.1 mm for body length, wing length, tail length and tarsus length. After the measurements and blood sample collections, the parent birds were released, and the nestlings were returned to their original nest boxes.

### Breeding density and synchrony

We expressed local density as the number of nests within 700 m (the median distance that we observed between a sire and his extra-group offspring, where breeding birds have a high chance of interacting with each other [60]). We measured local breeding synchrony as the proportion of simultaneous territories within 700 m, where the fertile period of a female overlapped with that of a focal female by one or more days. The local synchrony index was this number divided by the number of females occupying boxes within the territory [61]. In most passerine birds, the average duration of the fertile period is known to have an average duration of viable sperm storage of 8 days [62]. Accordingly, the female’s fertile period was defined as day -4 to day +3 of the breeding cycle (day 0 is the laying date of first egg), which was chosen as a conservative estimate of a passerine’s fertile period [63].

### Microsatellite genotyping

Only nests including the two social mates and the whole brood at 6–7 d were used to estimate extra-pair paternity. Adult and nestling DNA was extracted from blood samples using the UNIQ-10 column animal genomic DNA isolation kit (SK1206, Sangon, Shanghai). We assigned parentage to all offspring by genotyping all nestlings and candidate parents. Samples collected in 2011 and 2012 were typed with nine highly polymorphic microsatellite loci: Fhy341, Fhy226, Fhy428, Fhy429, Fhy310, Fhy415, Fhy344, Fhy444 and Fhy450 [64]; samples collected in 2013 were typed with ten highly polymorphic microsatellite loci: Fhy463, Fhy341, Fhy321, Fhy429, Fhy458, Fhy415, Fhy453, Fhy344, Fhy444 and Fhy450 [64]. PCR products were run on an ABI PRISM 3100 Genetic Analyzer (Applied Biosystems, Foster City, CA, USA) with the GeneScan 500 ROX size standard.

### Paternity analyses and identification of extra-pair males

We used CERVUS 3.0 [65] to calculate allele frequencies, heterozygosity values, exclusion probabilities and deviation from Hardy-Weinberg equilibrium based on the genetic data of all adult and nestlings, and the maximum likelihood method in the program was adopted to assess offspring parentage. CERVUS calculates a log-likelihood ratio (LOD score) for each parent that is a candidate for parentage of a given offspring. A high LOD score indicates that the candidate parent is likely to be the true parent, and a low LOD score indicates that the candidate parent is not directly related to the offspring. The simulation program (reiterated for 10,000 cycles) within the CERVUS was used to estimate the required critical difference in LOD (natural logarithm of the likelihood ratio) scores between the first and second most likely candidate sire for assignment at a > 95% confidence level. Positive log-likelihood ratio scores suggested that the candidate male was more likely to be the father than a randomly chosen male [5]. If none of the candidate males met this criterion, an unsampled individual (e.g., a non-resident ‘floater’ male) was considered to have sired the offspring. The autosomal microsatellite loci had a combined exclusionary power of 0.9989 for the first parent and 0.9999 for the second parent. We considered nestlings to be the offspring of the adults attending the nests when their genotypes were compatible with those of the males and females for the loci that were typed. Nestlings were categorized as within-pair if all their loci matched those of their candidate social father or if we found only one mismatch. They were considered extra-pair if their genotype mismatched their putative social father’s genotype at two or more loci.

### Statistical analyses

We calculated the standardized individual heterozygosity (SH) (proportion of heterozygous loci/mean heterozygosity of typed loci) as individual heterozygosity for simplicity because not all individuals were typed with the same set of microsatellite markers [66]. Genetic similarity between individuals was calculated as pair-wise relatedness values (r) using the method of Queller and Goodnight (1989) [67], which was implemented in KINGROUP v2 [68]. This score is a likelihood estimate of relatedness based on gene sharing (a score of -1 represents two maximally dissimilar individuals, a score of 1 indicates clones, and a score of 0 represents the average relatedness of two randomly chosen individuals in the population).

A generalized linear mixed model (GLMM) with binomial error distribution was used to test the effect of breeding synchrony index and local breeding density on the occurrence of extra-pair paternity. We performed independent-sample t-tests to examine differences in heterozygosity between females that produced extra-pair young and females that produced only within-pair young, together with the relatedness of pairs that produced extra-pair young and pair bonds that produced only within-pair young. Paired t-tests were also used to compare the heterozygosity and male phenotypic characteristics (e.g., bill length, tarsus length, wing length, tail length and body length) between cuckolded and cuckolder males (n = 34) as well as between cuckolded males and their neighboring males (n = 30). We also used paired t-tests to compare the heterozygosity of extra-pair young and within-pair maternal half-siblings within mixed paternity broods (n = 30). Spearman’s pairwise test was used to analyze the relationships between male phenotypic characteristics and heterozygosity (n = 64).

Statistical analyses were performed in R version 3.3.2 (R Development Core Team, http://cran.r-project.org/) [69]. All tests were two tailed with a significance level of p < 0.05. The values are expressed as the mean ± SE throughout.

## Results

### Pattern of extra-pair paternity

We analyzed the paternity of 325 nestlings from 64 broods, and we found that 54.7% of nests (n = 35) contained at least one extra-pair young (5 nests whose genetic fathers were not determined), and 22.15% of the offspring (n = 72) were determined to be extra-pair young. A total of 76.4% (n = 55) of the extra-pair young from 30 nests were assigned to the genetic father. The number of extra-pair young per brood varied from one to four, and the number of genetic fathers ranged from one to two.

### Nearest-neighbor distances and ecological factors

Most female yellow-rumped flycatchers (31 out of 35 nests) selected the non-nearest neighboring males as extra-pair mates; the mean ± SE distance between nests of extra-pair sires and the cuckolded males was 714.89 ± 94.74 m (n = 34, range 44.33–1767 m), and the distance between the nests of cuckolded males and their nearest neighbors was 173.11 ± 17.91 m (n = 31, range 40.53–370.18 m). Four cases of extra-pair copulations occurred between the nearest neighbors

Within our study population, the presence/absence of extra-pair paternity was not related to breeding synchrony (GLMM: Z = 1.175, p = 0.240), local breeding density (GLMM: Z = 0.604, p = 0.546) or their interaction (GLMM: Z = -1.288, p = 0.198).

### Individual genetic and phenotypic characteristics

The body length of cuckolders was significantly larger than that of males that were cuckolded (paired t-test: t_34_ = -2.218, p = 0.034), while no differences were detected between cuckolded males and their neighboring males (Table 1).

**Table 1:** Comparisons of phenotypic characteristics between cuckolder and cuckolded males (pairwise comparisons) (n = 34), cuckolded males and their neighboring males (n = 30).

	Male status			Cuckolder versus cuckolded		Cuckolded versus neighbor	
	Cuckolded (n = 35)	Cuckolder (n = 27)	Neighbor (n = 31)	t	p	t	p
Bill(mm)	7.88 ± 0.09	7.91 ± 0.12	7.93 ± 0.11	-0.150	0.882	-0.175	0.862
Tarsus (mm)	18.92 ± 0.17	19.15 ± 0.15	18.98 ± 0.16	-0.806	0.426	0.633	0.532
Wing (mm)	68.04 ± 0.44	67.99 ± 0.56	68.51 ± 0.55	1.525	0.137	0.034	0.973
Tail (mm)	43.00 ± 0.50	42.97 ± 0.56	43.47 ± 0.62	0.764	0.450	-1.038	0.308
Length (mm)	116.93 ± 0.79	118.94 ± 0.87	117.20 ± 0.84	-2.218	0.034	0.710	0.483

**Fig 1:**
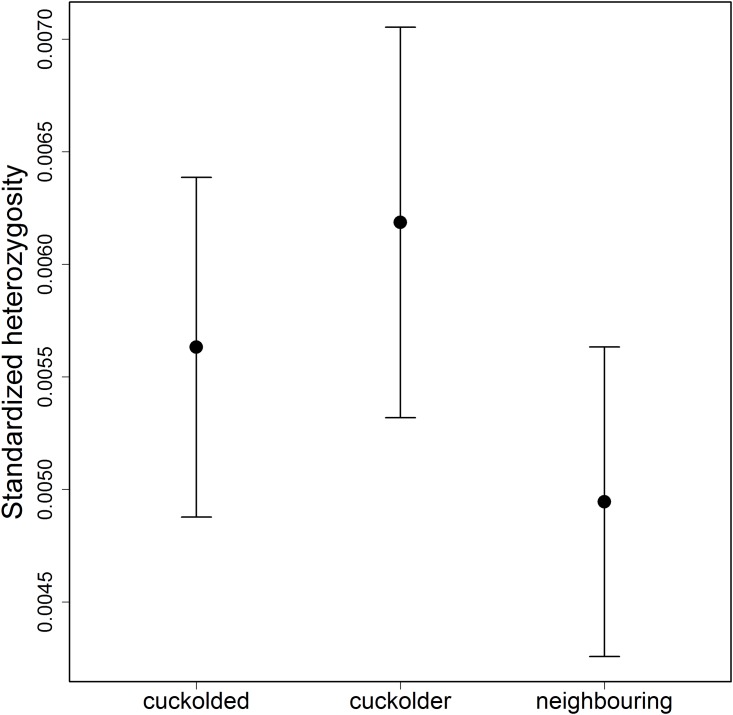
Standardized heterozygosity for cuckolded males and their nearest neighbouring males, and cuckolder males. The y-axis represents the standardized heterozygosity, and the x-axis represents the male status. Results are presented as means ± SE, and upper-lower 95% confidence intervals for each group were: cuckolded males: 0.0056 ± 0.0007 (n = 35), cuckolder males: 0.0062 ± 0.0008 (n = 26), neighbouring males: 0.0049 ± 0.0006 (n = 28).

**Fig 2:**
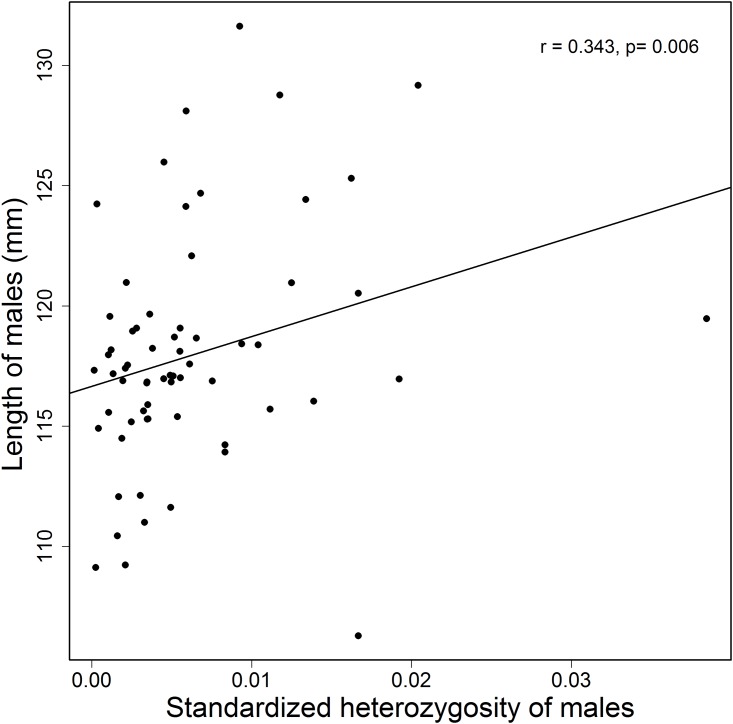
Correlation between standardized heterozygosity and body length of male yellow-rumped flycatchers. Small dots represent one data point. Lines are based on least-squares regression. Body length and standardized heterozygosity, Spearman rank correlation coefficient: r = 0.343, p = 0.006, n = 64.

The cuckolders were more heterozygous than cuckolded males (paired t-test: t_34_ = -2.419, p = 0.021) but the cuckolds did not differ from the neighbors of cuckolded males (paired t-test: t_27_ = -1.770, p = 0.088) (Fig 1). Moreover, there was a significant positive correlation between male heterozygosity and body length (Spearman’s correlation: r = 0.343, n = 64, p = 0.006) (Fig 2).

Social mates of broods containing extra-pair offspring did not differ in genetic similarity from pairs without extra-pair offspring (sample t-test: t_35, 29_ = 0.226, p = 0.822). The extra-pair and social males did not differ in relatedness to the females (paired t-test: t_34_ = 0.644, p = 0.524). In addition, within mixed paternity broods, extra-pair young were more heterozygous than their within-pair maternal half-siblings (paired t-test: t_30_ = 2.248, p = 0.032).

## Discussion

Two hypotheses have been proposed for explaining the indirect genetic benefits that a female would gain through her choice of a mate. The good genes hypothesis postulates that females will benefit from copulating with high-quality extra-pair males by producing extra-pair offspring with enhanced genetic viability, assuming that females can assess the quality of potential mates based on heritable male characteristics that honestly reflect quality, such as body size and ornaments [11,70]. The genetic compatibility hypothesis posits that females choose extra-pair males based on genetic dissimilarity [6,71], assuming that the fitness of an offspring is positively correlated with its heterozygosity [72]. Compatible gene effects can occur in several ways, including female preferences for mates that are dissimilar [73] or relatively heterozygous in general (e.g., inbreeding avoidance) [16]. In this study, we found that the selection of extra-pair mates was not random in the yellow-rumped flycatchers, females tended to choose larger and more heterozygous males than their social mates as extra-pair mates, and male body length was positively correlated with heterozygosity (Fig 2). Previous studies have found positive correlations between heterozygosity and various fitness-related traits such as survival, territory size, clutch size, fertilization, hatching and fledging success [72], and correlations between heterozygosity and condition dependent phenotypic traits [16,74]; heterozygosity preferences could be used as a quality trait in mate choice [6,75,76]. And heterozygosity has been associated with higher offspring survival rates [77,78], disease resistance [79] and developmental stability [72]. We found the relatedness of pairs that produced extra-pair young did not differ from pair bonds that produced only within-pair young, and extra-pair offspring were more heterozygous than their within-pair half-siblings in the yellow-rumped flycatchers. However, in view of high heterogeneity of effect sizes [80], potential sampling bias due to non-random offspring mortality prior to sampling [81], inconsistent definitions of EPP [82], and evidence that inbreeding maybe only problematic beyond an extreme threshold value or at particular loci [83], adequate statistical and molecular methods must be applied when estimating the relationship between EPP and genetic relatedness. Therefore, our findings support the good genes hypothesis but do not exclude the compatibility hypothesis.

The occurrence of extra-pair copulations requires that a female encounters an extra-pair male and copulates successfully. In passerine birds, a high breeding density may give individuals more opportunities to encounter and compare extra-pair mates. In addition, breeding density may influence the effects of breeding synchrony on extra-pair paternity rates by altering the proximity of fertile females or of neighboring males, which increase the occurrence of extra-pair copulations [2,63] and consequently, close neighboring males are most likely to be extra-pair fathers [25,39,41]. In this study, we found that most female yellow-rumped flycatchers (31 out of 35) attained extra-pair paternity with distant males rather than their nearest accessible neighboring males. Because many other hypothesized factors may affect extra-pair paternity, positive interaction between synchrony and density has not been detected in all species where it has been tested, and no evidence has been found for a main effect of either variable [84,85]. For the yellow-rumped flycatchers, cuckolded nests were surrounded by an average of 8.4 neighbors within a 700 m radius (at least 3 neighbors in breeding synchrony). The mean distance between nests of cuckolded males and their nearest neighbors was approximately 173 m, and breeding density, breeding synchrony and their interaction did not affect the occurrence of extra-pair paternity in the species. Furthermore, there was little variation in ecological factors (e.g. habitat heterogeneity) within the study population. In some species, mate guarding is a strategy to avoid extra-pair mating, and the males always show aggression towards intruders especially for nearest neighbors [26]. However we did not find any cases of aggressive behavior between male-male or male-female (unpublished data) pairs in the population. Thus, there may be other reasons why females seldom attain extra-pair paternity with the closest accessible males in the species.

In territorial birds, the formation of a conspecific neighborhood is generally a result of competition for space, food and mates [34,82], and it is conceivable that higher quality males may force weaker competitors to withdraw to suboptimal nest sites through dominance interactions [86,87], coexist with equivalent competitors, or tolerate low-quality males settling nearby [34]. Different neighborhood compositions might affect the distribution of extra-pair paternity. For example, Formica et al (2003) found that in white-throated sparrows (*Zonotrichia albicollis*), high-quality males tend to settle in high-density areas, where the probability of encountering neighboring fertile females is greatest [86]. In lazuli buntings *(Passerina amoena)*, high quality males permit dull yearling males to settle nearby, which can increase their opportunities to obtain extra-pair copulations [34]. Studies have demonstrated that females prefer dominant individuals for extra-pair copulations to enhance offspring fitness [87,88]. Therefore, distance between social and extra-pair nest may be a function of the quality of extra-pair males. For example, females that mated with high-quality social partners, or are surrounded by close male relatives will likely need to travel farther away to find extra-pair males [31,54,55]. In yellow-rumped flycatchers, we found that extra-pair males were larger and more heterozygous than the corresponding within-pair males, but no differences were detected between nearest neighbors regardless of whether they were being cuckolded or not. Moreover, no close male relatives were recorded as being immediate neighbors according to banding (unpublished data). Thus, we suggest that a low level of extra-pair paternity between immediate neighbors might result from female preferences for high-quality males in the flycatcher.

In most long-distance migratory passerines, early-arriving birds are often higher quality individuals or are in better condition [89], and they occupy relatively good territories and obtain more mates or higher quality mates [89–91]. Early-breeding males usually pursue extra-pair copulations after the onset of their social mate’s egg laying [92] because they might have already solved the conflict over paternity [93]. Paternity success in a male's own brood may depend on the efficiency of its paternity assurance strategies and its social mate’s willingness to engage in extra-pair copulation [94]. Under such conditions, actively selecting a low-quality male as the nearest neighbor would reduce the willingness of a male's social mate to gain extra-pair copulations from its rival. This idea warrants further study using experimental manipulations to test the hypothesis properly.

## Supporting information

S1 Table Summary of the raw data in “Microsatellite loci, phenotypic characteristics and breeding parameters of yellow-rumped flycatcher”. (XLSX)
